# A Baby Named Louise: Medical Ethics, Informed Consent and the Value of Disabled Infant Lives in the Summer of 1978

**DOI:** 10.1093/shm/hkaf033

**Published:** 2026-03-06

**Authors:** Vicky Long

**Affiliations:** Twentieth-Century British History, School of History, Classics and Archaeology, Armstrong Building, Newcastle University, NE1 7RU, UK

The birth of Louise Brown, the first baby conceived through in vitro fertilisation (hereafter IVF), at Oldham General Hospital in July 1978, attracted extensive media interest. The historical significance of Brown’s conception and birth is reflected in the body of scholarly literature on this subject, and the archiving of related materials.^1^ Brown’s story serves as a comparative lens to examine responses to another baby named Louise, who was born in month after Brown, at High Wycombe Hospital in England, and died a few weeks later. Like Brown, this Louise’s story was also broadcast on television. However, while Brown’s story still features in popular media coverage and academic scholarship, this other Louise’s tale is now largely invisible. In this article, she remains Louise; I will use Louise Brown or Brown to indicate her more well-known namesake. Resurrecting Louise’s story extends efforts to write babies into history as subjects,^2^ revealing how attitudes towards age and disability shaped emerging practices of informed consent and the nascent medical ethics movement. Louise’s story adds new insights into existing studies of the development of bioethics,^3^ revealing how early attempts to devise an ethical approach to caring for babies born with congenital anomalies wrought harm on parents, babies and doctors.

I start by examining how to locate babies who lived short lives as subjects within our research, before re-examining Louise Brown’s story through the lens of disability. I then examine how developments in treatment for spina bifida myelomeningocele, the congenital anomaly Louise was born with, significantly reduced morbidity and mortality. I chart how some doctors began to argue that only the most clinically promising cases should be actively treated and examine what ‘selective non-treatment’ entailed in practice. Focussing on two documentaries about Louise’s case, I then explore the dilemmas facing doctors and parents of babies born with spina bifida and other congenital anomalies in an era where there was no medical consensus about the most appropriate treatment, revealing the often harrowing, long-term impacts of nominally involving parents in decision-making. Parents’ and healthcare workers’ divergent views about the value of disabled children’s lives are then examined, highlighting how the dearth of adequate welfare provisions contributed to perceptions that disabled children imposed a burden on their families. Finally, I explore how obfuscation surrounding the care of babies who were not actively treated, tacitly endorsed by the Department of Health and Social Services (DHSS), failed to prevent public scrutiny or stop the police from opening criminal investigations. It did, however, halt investigations into doctors from proceeding to prosecutions for murder or manslaughter.

## Two babies named Louise

Louise Brown’s birth in 1978 to Lesley and John Brown represented the culmination of years of research into IVF and embryo transfer.^4^ The circumstances of Louise Brown’s conception attracted considerable criticism, and her birth and infancy came under intense public scrutiny.^5^ Criticism of the ethics of IVF has declined as the practice has become normalised, but public interest in Brown remains strong: the 40th anniversary of her birth in 2018 was widely marked as a celebratory event,^6^ while YouTube streams video footage of her birth.^7^

Conversely, I cannot establish some of the basic facts about Louise. She was born in August 1978 and died three weeks later, but I cannot pinpoint her date of birth or death. Thames Television and ATV, which broadcast to London and the Midlands respectively, aired two programmes about Louise in January 1979, produced within Thames Television’s *Afternoon Plus* series. The BFI does not hold footage for the two episodes of Louise, and only the opening 30 seconds, and an advert break, can be found online.^8^ I located the transcripts following a freedom of information request to open a public prosecution file that I deduced referred to the case.^9^ To comply with data protection requirements, Louise’s surname was redacted.

How can we approach historical subjects like Louise, whose lives stretched to mere days and weeks, and what value is there in trying to centre such subjects in our research? It is difficult to use the sources historians typically exploit to examine children’s agency, when studying babies who cannot write, speak, or purposively exert agency. Yet even newborn babies communicate. Take the harrowing testimony from the mother of murdered baby ‘E’ at the trial of serial killer Lucy Letby. She described how her son cried ‘like nothing I had heard before… It was a sound that shouldn’t have come from a tiny baby.’^10^ Her testimony highlights her son’s capacity to feel and express pain, and his lack of agency. Acknowledging that babies were ‘inarticulate historical actors’ rather than agents, Janet Golden suggests that we focus on the interactions between individual babies’ lives and actions taken by adults on behalf of babies.^11^

Baby E’s mother entered details of the assault he experienced into the historical record, revealing the impact of his short life and death on her, and the widespread interest generated by his murder. We might similarly acknowledge Louise’s lack of agency while affirming her personhood and capacity to feel, and recognise that her life and death affected her family. I will place Louise at the heart of this analysis by examining her life and death, the actions taken by adults around her, and the wider ramifications of those actions. Where children’s voices and actions are absent from the historical record, we must listen to adults’ reports. However, such accounts rarely foreground children’s perspectives.^12^ Some proponents of selective non-treatment claimed that babies’ and parents’ interests were intrinsically entwined, grounding this claim on understanding of infant development and attachment theory. Yet, attachment theory was mobilised to justify withholding care from disabled infants, concealing how parents’ and children’s interests might diverge, and failing to acknowledge that power in familial relationships rested in parents’ hands.

The stories of the two Louises diverged because of how Brown was conceived, and Louise’s disability. However, the spectre of congenital anomalies influenced physiologist Robert Edwards and gynaecologist Patrick Steptoe, whose research into IVF led to Brown’s birth. Indeed, Edwards only focussed on infertility after meeting Steptoe in 1968, having previously researched the causes and prevention of genetic anomalies.^13^ Both men knew that support from medical colleagues and the media was contingent on being able to demonstrate the safety of IVF. Anxieties that IVF might cause birth defects hindered their research and underpinned many objections from medical and lay commentators. A 1971 application for Medical Research Council funding was rejected after reviewers expressed concerns that IVF might cause congenital anomalies, for example.^14^ The geneticist and biophysicist James Watson claimed Edwards and Steptoe’s work necessitated acceptance of infanticide, as ‘there are going to be a lot of mistakes’, while biologist and bioethicist Leon Kass similarly asserted that IVF would cause deformity.^15^

In her autobiography, Brown comments ‘quite what would have happened to the future of IVF if I had been born with any kind of disability or defect is hard to tell’.^16^ She hypothesises that had anything been ‘wrong’ with her, critics could have blocked further IVF procedures. Edwards largely dismissed criticisms that IVF could lead to abnormal foetal development. Amniocentesis, a method to prenatally diagnose such anomalies,^17^ served as a ‘well-proven line of defence’ against ‘chromosomal disasters’.^18^ Edwards and Steptoe would only implant an embryo after the Browns signed a document permitting Steptoe to undertake an amniocentesis, and to abort the baby immediately if it was ‘abnormal in any way’.^19^ Louise Brown describes how more than 60 tests were undertaken on her during her first days, to prove ‘to a sceptical world that creating a baby in this way did not cause any problems or abnormality’.^20^ The Browns’ deal with the Associated Newspaper Group for exclusive coverage of their daughter’s birth included a clause that the full sum would only be paid if the baby was not ‘significantly malformed or mentally retarded’.^21^ Indeed, even Brown’s body could not escape intense public scrutiny, and she appeared naked in a 1978 Thames television documentary, *To Mrs Brown… A Daughter*, to prove that no anomalies were being concealed.^22^ Brown’s story, then, reveals how disability in general, and congenital anomalies in particular, were viewed by many in the late 1970s as a disaster to be avoided at all costs.

## The treatment of spina bifida in twentieth-century Britain

To understand Louise’s story, knowledge of ableism and societal discrimination, while important, is insufficient. We also need knowledge of myelomeningocele, and how medical and surgical treatment evolved. Historians of medicine may not question this, but as the social model of disability asserts that people are disabled by societal barriers, not impairment, the point is worth addressing. Tom Shakespeare and Nicholas Watson contend that disability is ‘a complex dialectic of biological, psychological, cultural and socio-political factors’; it cannot be defined purely as impairment, but should not be attributed solely to societal barriers.^23^ This approach is helpful when analysing the history spina bifida myelomeningocele, described in a recent surgical guide as ‘one of the most devastating congenital malformations that are compatible with life’.^24^ Spina bifida occurs when the neural tube fails to close in early pregnancy. Neural tube defects diverge in prognosis depending on their location and size. Anencephaly, where the top of the tube remains open, is characterised by the absence of most of the brain, scalp and skull; affected babies usually die during or shortly after birth. Conversely, spina bifida occulta, in which some of the vertebrae have not fully closed but the spinal cord and nerves are usually unaffected, may go undetected. In spina bifida meningocele, a sack protrudes from the back containing fluid. Myelomeningocele, the more common form of open spina bifida, denotes cases where part of the spinal cord and nerves are in this sac and are damaged, causing more impairment. The impairments caused by myelomeningocele can affect movement, bowel and bladder function, skin sensation and in some cases learning and development. The degree of impairment depends partly on the size and location of the lesion, the extent of nerve and spinal cord damage, and the presence or absence of hydrocephalus—a build-up of fluid in the brain—which can cause brain damage and death. Hydrocephalus is a common complication of spina bifida.

Historically, most babies born with spina bifida myelomeningocele died in infancy from renal failure, infections such as meningitis, and hydrocephalus. Medical and surgical developments between the 1940s and mid-1960s transformed myelomeningocele from a usually lethal to a frequently survivable condition. The first of these developments was earlier, more systematic post-birth surgical closure of the lesion, pioneered by the neurosurgeon Franc Ingraham at Boston Children’s Hospital in the late 1930s and early 1940s.^25^ Hydrocephalus became easier to control with shunts that removed excess fluid from the brain following the development of the Spitz-Holter valve in 1956, which proved less failure-prone than earlier ball valves.^26^ Finally, the development of effective antibiotic treatments reduced meningitis mortality,^27^ a common complication of spina bifida. At Sheffield’s Children Hospital, a team including paediatric surgeon Robert Zachary, who had trained at Boston with Ingraham,^28^ and the paediatrician John Lorber, showed that surgical repair within 48 hours of birth resulted in lowered mortality, shorter hospital stays, lowered incidence of meningitis and sepsis, and reduced paralysis. They concluded that immediate operative closure of myelomeningocele led to the best clinical outcome.^29^ New specialist regional units emerged in NHS hospitals, where paediatric and orthopaedic surgeons, urologists and paediatricians collaborated to save babies’ lives and minimise their impairments. Many survivors had numerous operations to repair the myelomeningocele, install and revise a valve to control hydrocephalus and remedy scoliosis, a curvature of the spine. A 1964 Ministry of Health briefing note suggested the survival rate among babies born with myelomeningocele had increased from 33 to 50–70 per cent following the introduction of rapid surgical closure, with the potential to improve further.^30^

Paediatricians and paediatric surgeons in this era had more capacity to treat congenital anomalies due to the substantial decline in infectious diseases, following the introduction of mass vaccinations.^31^ Yet regional specialist units developed unevenly. Surgeons wrote to the Chief Medical Officer in the 1960s to plead for enhanced neonatal surgical facilities, describing overcrowding which fuelled cross-infection and mortality, and turning patients away who would die as a result.^32^ Under pressure from these clinicians, the Standing Medical Advisory Committee, which provided clinical advice to the government on NHS provisions, established a working party. Its 1968 report urged regional hospital boards to establish and expand provisions and train more paediatric surgeons.^33^ The report unequivocally stated there was ‘rarely justification for adopting a passive attitude towards any deformed newborn baby who has a greater chance of survival than in the past’ and that ‘everything that is practicable should be done.’^34^

A few years later, this certainty dissolved into a heated debate: should babies born with myelomeningoceles be actively treated or ‘encouraged’ to die? When John Lorber, who helped pioneer early surgical repair in the UK, publicly broke from his former collaborator Zachary to claim that spina bifida survivors caused ‘suffering, deprivation, anxiety, frustration, family stress and financial cost’ for patients, their families and the community,^35^ the DHSS willingly embraced his change of heart. It issued *Care of the Child with Spina Bifida* in 1973, encouraging doctors to consider only treating the more clinically promising cases.^36^ Decisions about ‘early surgical treatment, and subsequent medical management are the responsibility of the individual doctor concerned,’ the guidance stated, noting ‘some workers are now trying to apply a policy of selection’ to decide which babies should be actively treated.^37^ Similar debates occurred in the United States, though triggered by different cases and research studies.^38^

While the DHSS tacitly endorsed selective treatment, it did not outline how babies not selected for active treatment should be cared for. Doctors rarely detailed care regimes for untreated babies but piecemeal evidence indicates the breadth of practices adopted under the heading ‘selective non-treatment’, which led to divergent survival rates. This stretched from withholding surgery but actively treating infection and hydrocephalus and ensuring adequate nutrition, to intentionally causing the deaths of babies who might otherwise have survived by dosing heavily with sedatives and restricting food intake. Lorber claimed ‘the overwhelming majority of untreated infants with myelomeningocele die early in life’, but this depended on the care regime. Babies, he admitted, would ‘live longer’ if provided with oxygen, tube feeding, antibiotics and skilled nursing care. He advocated ‘“custodial care”: ordinary feeding and nursing procedures, but no more’.^39^

A team from Liverpool’s Alder Hey Children’s Hospital reported that many babies whose lesions were not repaired would survive if provided with a shunt to manage hydrocephalus.^40^ Among a cohort of babies born with myelomeningocele at North Staffs Hospital between 1971 and 1981 who received neither shunt nor surgical repair, the maximum survival duration was 240 days. These researchers found that home as opposed to hospital care correlated with a longer lifespan. Wendy, the longest survivor, was brought home by her mother against the advice of the hospital. We cannot know whether antibiotics would have prevented Wendy’s death from meningitis, though Wendy’s mother expressed regret for giving her daughter chloral, only discovering later that it probably hastened Wendy’s death.^41^

In 1977, Zachary attacked the ‘myth’ that babies would ‘all die spontaneously’ if not operated on. He observed that babies were often given four daily doses of chloral hydrate, each of which exceeded eightfold the dose recommended for sedative purposes in the standard paediatric handbook. ‘No wonder these babies are sleepy and demand no feed’, Zachary wrote. ‘Most of them will die within the first few weeks, many within the first week.’ Describing a centre where all unoperated patients died, Zachary recalled how the question, ‘did they fall or were they pushed’ [into death]? met with the answer, ‘they were pushed of course’. On another occasion, Zachery remembered a paediatrician responding to a query about how babies with myelomeningocele were managed with the words, ‘we don’t feed them.’^42^ The American paediatrician John Freeman reported that untreated babies did not die quickly, attributing the rapid deaths in Britain to overuse of phenobarbital and restricted feeding, causing deaths by starvation.^43^ Norman Guthkelch, Britain’s first professor of paediatric neurosurgery, later recalled how doctors’ opinions ranged from ‘maximal care’ to those ‘who supported what amounted to euthanasia.’ He blamed the government for failing to address ‘these difficult issues’, instead delegating this ‘to the discretion of individual practitioners, with highly variable results.’^44^

## Televising Louise’s Life and Death

The treatment of Louise was by no means unique. She received an operation shortly after birth to repair her myelomeningocele but died after her parents and paediatrician Dr Donald Garrow decided to withhold further treatment and add sedation to her bottle feeds. It was, however, unusual for an individual case to reach public attention, and for doctors to openly discuss and rationalise their role in the deaths of such patients. The *Afternoon Plus* programmes, aired in January 1979, brought to public view a debate which most doctors were reticent to discuss in published work. Indeed, of the three doctors who participated in the programmes, only Zachary had published explicitly on this topic.

*Afternoon Plus*, sometimes listed as *After Noon Plus*, aired between 1979 and 1982. It was part of the longer lineage of daytime magazine programming produced by Thames Television between 1971 and 1988, under different titles.^45^ ATV and Thames aired the episodes on Louise’s case: these two independent television regional franchises covered more than 36 per cent of UK homes in 1980.^46^ There is no central ATV or Thames Television archive. Some written materials are held in the Independent Broadcasting Authority Archive at Bournemouth, and within the BFI archive, but catalogue searches do not reveal anything relating to these episodes.

*Afternoon Plus* dealt with only one or two topics, selected on the interests of that episode’s presenters, and varied in format from studio discussions, one-on-one interviews, sofa discussions, and investigative films.^47^ Jilly Boyce Kay argues that the limited scholarship on *Afternoon Plus* reflects the low cultural status associated with ITV, and daytime programming for women.^48^ Youtube snippets from the programme—typically, high profile interviews and cookery sections—reinforce perceptions that the programme’s contents squarely targeted women. Producers and presenters aimed at a wider audience, and many episodes addressed social and political issues, including abortion, contraception, divorce and mental health.^49^ The episodes on Louise’s case, presented by veteran actor and TV and radio presenter Bernard Braden,^50^ fall within this category.

The episodes sat within a wider body of television programming in the 1970s and 1980s that was critical of medical developments and practices, or examined ethics in medicine.^51^ Given the dearth of contextual information on the episodes relating to Louise’s case, I cannot analyse audience responses, as Elizabeth Toon does for the 1975 television play on breast cancer, *Through the Night*.^52^ The episodes did not redefine television coverage of medical issues, as the 1968 *Tomorrow’s World* studio debate on heart transplant operations had done.^53^ Nor was this the only television or radio coverage on the ethics of selective non-treatment.^54^ Nevertheless, there are compelling reasons for analysing these episodes. Anne Karpf observes that television programmes framed medical ethics as matters of individual conscience, ignoring the disparity of power between doctors and patients.^55^ These episodes allow us to examine the impacts of this at a granular level. Surviving records reveal how anti-abortion organisations and the police responded to selective non-treatment, revealing the consciously opaque set of legal and medical responses to these practices. Finally, as a daytime magazine show aimed primarily at women, the episodes provided a rare arena for mothers to share their experiences of their disabled child’s medical care, and whether they had felt able to influence this.

Ostensibly the programmes debated the ethics of actively treating babies born with myelomeningoceles by including doctors with divergent perspectives. However, in opening the programme, the lead presenter Bernard Braden suggested that Louise’s case raised important questions about ‘whether babies born with particularly severe abnormality should be allowed to die rather than suffer for the rest of their lives’.^56^ Although Garrow hastened Louise’s death by adding sedation to her bottles and withholding treatment for meningitis, in Braden’s turn of phrase Louise was not helped to die, and certainly was not killed; rather, those involved had simply chosen not to misguidedly try to prevent her death. And morally, Braden suggested this was the best course of action to take because life with spina bifida inevitably entailed suffering. Garrow later echoed Braden’s presentation of the issue when he outlined the two different approaches, which he termed active treatment, and letting ‘nature take its course,’ only providing a moral rationale for the latter. ‘There is quite a body of opinion now, that accepts that this condition which may be very severe and be associated with very severe deformities, is more than can be tolerated’, he claimed. Garrow presented disabled people’s dissatisfactions as a product of their impairments, failing to acknowledge that discriminatory attitudes towards disability affected the quality of life. To undertake active treatment, he argued, was ‘to condemn someone to a life of repeated hospital visits, and discomforts, and unhappinesses’.

Knowledge of the doctors who participated in the programmes sheds light on the factors that shaped their views. Garrow, the paediatrician in charge of Louise’s care, led the paediatric department at High Wycombe Hospital from its opening in 1962, until his retirement in 1983. He previously worked at Amersham Hospital alongside Dermod MacCarthy, a keen advocate of liberalising hospital visiting for children.^57^ Garrow, in turn, also became known as an advocate for parent–infant attachment and influenced the design of Wycombe Hospital’s special care baby unit, which had rooms that enabled women to be admitted with their babies.^58^ Garrow felt it was ‘wrong and unnecessary’ to separate mothers from their infants, concluding that neonatal separation impeded the development of maternal feelings and had long-lasting consequences. Mothers, he claimed, reported feeling upset and remote from their babies when separated from them, often stating of their babies, ‘I felt he wasn’t really mine until I got him home.’^59^ Garrow therefore consciously aimed to restrict maternal attachment when he encouraged Louise’s mother to return home without her. In a later documentary, he likewise encouraged the mother of Nicola, a baby born with a posterior encephalocele—a neural tube defect where the skull has not fully formed—to do the same.^60^ Perhaps he hoped to minimise the emotional impact of their babies’ deaths, but equally, he may have aimed to dissuade them from taking their babies home or otherwise challenge his suggested approach to their babies’ care.

Zachary, a leading proponent of surgical repair of myelomeningocele, served as president of the Hydrocephalus Research Society from 1964 to 1957, and first chair for the Association for Spina Bifida and Hydrocephalus, which developed out of regional parent support groups and functioned as a national support association.^61^ He remained unwaveringly committed to actively treating babies with myelomeningocele, and vocally opposed Lorber’s view that only a minority should be selected for active treatment.^62^ His beliefs were influenced by his Catholic faith and his own experience of disability: Zachary was born with a severe curvature of the spine which made him feel that he shared a common bind with his spina bifida patients.^63^

Dr Claus Newman, the third doctor featured in the studio debate, was appointed in 1969 to Queen Mary’s in Roehampton to care for children born with thalidomide-related congenital anomalies. He later served as a medical advisor for the Thalidomide Trust. Thalidomide was prescribed for morning sickness in Britain between 1958 and 1961, and more than 400 children were subsequently born with limb and organ deformities.^64^ Recalling initial medical responses to the births of babies affected by thalidomide, Newman observed that ‘to begin with they were not expected to live… many were simply allowed to die.’^65^ The crisis did not prompt the establishment of welfare provisions for disabled people more broadly, because a compensation scheme for victims was established and no more people were affected by the drug following its withdrawal in 1961.^66^ However, the scandal raised awareness of families’ roles in caring for disabled children.^67^

## Parents, decision-making and informed consent

We turn now to the *Afternoon Plus* transcripts on Louise’s case. Louise’s birth and death, and debates about the morality and legality of withholding life-sustaining care, unfolded alongside growing public interest in and discussion of medical ethics. Describing his experiences as a paediatric registrar in the early to mid-1970s, Neil McIntosh recalls how ‘benevolent paternalism’ led him to turn babies’ ventilators off in the night and tell their mothers in the morning, to avoid disturbing their sleep.^68^ By the end of the decade, this approach was increasingly challenged, and the British medical profession began responding to calls for ethical guidelines. The British Paediatric Association established an ethics advisory committee in 1979,^69^ while the British Medical Association’s first *Handbook of Medical Ethics*, published in 1980, asserted that parents ‘must ultimately decide’ whether ‘severely malformed infants’ should be treated, but that doctors should help parents make these decisions.^70^ As Duncan Wilson shows, the 1980s were the turning point for British medical ethics and bioethics, as vocal demands began to be made for other professionals’ involvement in developing medical standards and practices.^71^

In a 1985 book for lay readers, journalist Carolyn Faulder accused doctors who practised benevolent paternalism of lying to patients.^72^ Doctors, she argued, should respect patients’ autonomy; patients should be fully informed of what they were being asked to consent to, and should determine what they felt was in their best interests. In paediatrics, parents were tasked with undertaking this role on behalf of their babies. To highlight the dilemmas facing parents, Faulder cited the case of paediatrician Leonard Arthur. Arthur withheld all nourishment bar water from John Pearson, a baby born with Down’s syndrome in 1980, at the behest of John’s parents who did not want him to survive, and prescribed painkillers to hasten John’s death. Arthur was subsequently prosecuted for murder but acquitted in 1981, and the case influenced medical organisations’ decisions to develop clearer ethical guidelines. For Faulder, the case highlighted how parents trying to do the best for their child might endorse wholly different courses of action, from saving their child’s life to bringing about their death.^73^ To contemporary sensibilities, John’s case reveals how parents might advocate a course of action entirely counter to their child’s interests, who was powerless in the decision-making process. Faulder overlooked this issue and never referred to John by name.

Beyond the issue of whether parents’ and children’s interests converged, we should question whether doctors enabled parents to make informed decisions about their child’s care. While Zachary agreed that parents should be involved in decision-making processes, he suggested what struck him as the most appropriate course of action and sought consent for this. Where the prognosis seemed good, Zachary viewed surgery as an urgent priority; conversely, he claimed he would advise parents against operating if survival prospects were very low. If an operation was possible, but the baby was completely paralysed, Zachary would give parents the option of the operation, or of dressing the lesion and ‘looking after the baby as well as any baby is entitled to be looked after in the 1970s’. However, he believed doctors should refuse to hasten a baby’s death through drugs.^74^ Zachary’s rather tokenistic involvement of parents in decision-making was doubtless shared by other doctors. The mother of a girl born with Down’s syndrome complained that her husband had been asked to consent to a lifesaving operation without being told her diagnosis. She thought Garrow was ‘absolutely marvellous’. She admitted though that had she been aware of her daughter’s diagnosis, ‘I don’t know what I would have done in the situation… but I would have liked the decision to be mine.’^75^

The information parents received from healthcare professionals often left much to be desired. Sometimes, it was extremely limited; Mary Lane’s mother, interviewed by researcher Caroline Glenndinning, felt the severity of her daughter’s impairment had been minimised: she was told that her daughter had to ‘go to Sheffield [the leading unit for surgical repair in the UK], she’s got a little lump at the bottom of her back.’ In other cases, health and social care workers might authoritatively pronounce a child’s prognosis, only to be proved wrong. A hospital almoner told Frances Molloy’s mother, ‘I want you to go home and just forget that she’s ever been born because she won’t live.’^76^ Frances was very much alive nine years later when her mother was interviewed, having been taken to Sheffield for surgical repair. Had she not received surgery, however, the original prognosis may have proved accurate.^77^ Shelly’s parents, meanwhile, were told that their daughter would ‘be a vegetable, that she would never survive an operation… we were told all the bleakest things’. Shelly was left without treatment for three weeks, before her parents moved her to a different hospital for an operation. They supported the principle of parents taking decisions, but ‘only after they’ve been told both sides of the story, which we weren’t’.^78^ While their decision diverged from the guidance they had received, they may have been in the minority. Indeed, they might not have contested the opinion of the doctor who originally treated Shelly, had her health declined rapidly.

Doctors’ own views about the merits of treating babies born with myelomeningocele and other congenital anomalies, and the quality of survivors’ lives, shaped their guidance. While parents were often led to believe they had a choice, doctors sought to influence them to make a particular decision. In the studio discussion, after several parents recounted how their now happy children had been written off by doctors at birth, Garrow insisted that he didn’t attempt to predict a child’s prognosis, aiming to ‘present both sides of the question’. However, earlier in the programme when explaining how he would ‘break the news to the parents of the deformity that their baby has, and… explain to the parents what this means in the long-term’, Garrow outlined every possible complication that could arise as a child born with spina bifida grew up. This must have been daunting for new parents with no prior knowledge of spina bifida to hear and was doubtless intended to sway parents to oppose active treatment, even though they were nominally offered a choice.

Eileen Delight and Janet Goodall, who interviewed parents of babies born with spina bifida who did not receive surgery some five to fourteen years after their babies’ deaths, found that parents remembered vividly the information doctors had provided. One set of parents recalled being told:

If an operation prolongs his life, he’ll never have any intelligence or know you. You’ll never be able to take him home and you won’t be able to visit him regularly if you have other children. It’s really for the best that he should die now.

As Delight and Goodall observed, people ‘staggering under the impact of statements like that cannot crystallise their own thoughts’.^79^ In an indirect rebuttal of Garrow’s approach, Zachary warned that parents were ‘given half the truth, they’re told the baby will not survive; the baby would be a vegetable… they should be told that not operating does not necessarily… mean that the baby’s going to die’. While Zachary agreed that surgeons needed parents’ permission to operate, he added, ‘let us not deceive ourselves into thinking they are making a decision which is not influenced by the doctor. A doctor… cannot but influence the patient, by the way he speaks.’^80^

Garrow asserted that parents who decided to withhold treatment ‘felt that it was the right decision’ and experienced ‘great relief that their child is not suffering’. Garrow told the interviewer that if parents asked for an operation and lifesaving care he would try and arrange this. However, noting that parents of another baby with spina bifida currently on his unit had decided to withhold treatment, Garrow commented ‘I’m glad about it, I think they’ve made the right decision.’^81^ What parents, facing unanticipated bad news and with no prior knowledge of the anomaly their baby was born with, would not want to make what the doctor deemed the ‘right’ decision? While Garrow valued parental involvement in decision-making in principle, he guided parents towards the decision he favoured. Writing elsewhere about his approach, he opined that ‘the effects of a handicapped child on other members of the family must be pointed out’, and that ‘to allow death to come… to a severely handicapped baby may be a seemly and loving thing to do’.^82^

Asked by the interviewer, ‘you were fond of her?’, Louise’s mother replied, ‘Well, we loved her, she was ours’. This won Garrow’s praise; Louise’s parents, he claimed, ‘were greatly to be admired’ for their care of their daughter. Louise’s parents may have unconsciously followed Garrow’s lead in how they should respond to their baby, given the lack of cultural scripts for acceptable parental behaviour in such circumstances, and perhaps to win his approval, as an authority figure. Garrow described their decision as ‘a really loving one’ which ‘wasn’t something they wanted for themselves’ but ‘something which they did for their child’. Louise’s parents initially decided to have the lesion surgically repaired, a decision Garrow supported as this could have prompted effective bladder and bowel functioning and little to no paralysis. However, following the operation tests indicated that Louise had little sensation in her legs, and she began to retain fluid, requiring manual expression of her bladder, which made Louise scream. Her parents’ desire to minimise Louise’s pain surfaced when they described the time leading up to her death, with Louise’s father commenting that she was ‘certainly in no pain’ and that ‘there was no way we wanted her to suffer’. Her parents were also daunted by the future surgeries Louise might require. As her mother explained, it didn’t seem fair ‘to let her have another operation just so that she could have more and more… it seemed silly to operate just so that she could suffer’. However, while Louise’s parents doubtless sought to make the best decision for her, wanted to minimise her pain and believed they should make those decisions as her parents, the emotional burden this imposed was very clear. As Louise’s mother explained, ‘We’ve got to feel we were doing the right thing, because if we didn’t we’d never live with it.’^83^

Annemarie Mol contrasts two models of healthcare: a logic of care, in which care is open-ended and dialogical, and a logic of choice, which asserts that patient choice is paramount, treatment is framed around decision-making, and is timebound.^84^ Doctors who advocated for parents to decide about their child’s treatment followed a logic of choice; care was withdrawn, the child died and the case was closed. Parents could go on to have other, healthy children who replaced the dead child. Yet if they harboured such a view, it was patently untrue. Paediatricians like Garrow typically did not follow up with parents after children’s deaths and would therefore be unaware of longer-term impacts. One father, interviewed ten years after the birth and death of his daughter, told the interviewer ‘There’s no way I’d be talking now if I hadn’t had a drink… there is no way you can get rid of that pain.’^85^ Mol notes that emotions unleashed by sudden illness can impair decision-making.^86^ These considerations are pertinent when considering parents who received unanticipated news about the health of their newborn baby with usually no prior knowledge about the congenital anomaly their baby had been born with. The pioneers of active treatment emphasised the importance of rapid surgical repair of spina bifida to optimise clinical outcomes, ideally within 48 hours of birth. This imposed an agonisingly short window of time for parents to make what was often framed as an irreversible decision about their baby’s treatment, even though surgical repair could occur later.

While Garrow emphasised the complications of spina bifida, and Zachary focussed on actively treating almost all babies, Newman spoke about the dilemmas facing doctors treating babies with ambiguous prognoses. ‘There is no clear criterion on which you can act’, he insisted. Having observed the ‘destructive effect of guilt’ experienced by parents of babies affected by Thalidomide, Newman felt parents should be spared the burden of decision-making. Rather than regale parents with a long list of every conceivable complication, Newman claimed that he preferred to take time to ascertain the specific outlook of each individual child and parents’ true feelings about their baby, although he did not clarify how he did this. If Newman felt the outlook was very bad and believed the parents shared this view and would prefer their child not to suffer any further, then he would not do anything further to help the child survive but would not share this decision with the parents. ‘I do not feel that the parents should share the guilt’, he told Braden.^87^

While this position ran counter to the emerging consensus that parents should participate in decision-making, it may have protected parents from long-term guilt. Among Goodall and Delight’s interviewees, parents who felt solely responsible for the decision to not operate on their child were most adversely burdened by guilt, and often dwelt on deficiencies in their child’s care. Unable to change the past and incapable of knowing whether different actions would have led to their child’s survival, all they could do was painfully re-examine why they had not insisted on different treatment at the time. They didn’t recognise how the privileging of medical expertise in hospital settings would have made it difficult to do so. Michael’s mother, who visited him in hospital, found that her son always seemed drugged, and wasn’t given enough milk; her interview suggests a misplaced sense of complicity in her son’s death. The nurses, she felt, did not leave enough time to feed him, and he gradually lost weight. ‘I was very angry because I was sure they were killing him by not feeding him enough’, she told the interviewer:

I kept having rows with Sister but she just said horrible things like, ‘He’s going to die anyway…it’s better he should die, otherwise he’ll be a burden to you’ – as if our son could ever be a burden to us! …we’d have done our best for him if had he lived – and he’d have lived much longer, perhaps even until now, if I’d have had him home. I would have made sure he had more food.^88^

## The value of disabled lives

The recollection of Michael’s mother highlights a prevalent view among health and social care workers at this time that a disabled child burdened their family, an attitude shaped by the earlier thalidomide tragedy. The Society for the Aid of Thalidomide Children, founded in 1962 by parents of affected children, claimed that with willpower, medical aid, and assistive devices these children could lead ‘normal’ lives. Most media coverage of and research into the families affected by Thalidomide, however, emphasised the burden imposed on parents and siblings, depicting the entire family as disabled.^89^ The notion that a disabled child would negatively affect able-bodied siblings, who would lose out on parental attention, could be mobilised to persuade parents to agree to non-­treatment, or to load guilt onto parents whose disabled child survived.

Some of the parents of surviving children who participated in the *Afternoon Plus* debate echoed this narrative, highlighting their hardships. One mother described Simon, her three-year-old son, who had cerebral palsy and was unable to talk, walk or crawl, as ‘an adorable little boy and very intelligent’. She told doctors after his birth that he should not be kept alive, but they disregarded her views and tube-fed Simon for three weeks until he could take a bottle. She felt he would become resentful when he discovered that he was unable to do what other people could. He attended a day school for disabled children, and she acknowledged that the children were ‘very happy’, but to her, the value of this was outweighed by the burden placed on parents with little respite from childcare.^90^ Another mother claimed her able-bodied children loved and adored their disabled brother, Peter, but insisted, ‘it doesn’t help family life to have a child so severely disabled’. Peter, she asserted, was ‘happy in the way our puppy’s happy’, a dehumanising analogy which suggests that she did not see her son as entitled to the usual protections afforded to people, reminding us that personhood was not automatically ascribed to disabled babies and children. Peter, in her view, ‘should not have been allowed to live… I hope to God he dies before he gets very much older’.^91^

Similar views were aired elsewhere by parents of disabled children. Twelve of fifteen mothers of adult children with learning disabilities interviewed in 1985, for example, opposed operating on babies born with congenital anomalies to prevent their deaths. ‘At the time I wanted him to live’, one responded, ‘but now realise this was a mistake… the strain of caring has been overwhelming’.^92^ The overwhelmingly negative views from this study cannot be dismissed, but the responses reflected the challenges of caring for disabled family members with little state support, and perhaps the stigma associated specifically with learning disabilities. The study’s design and findings likely also reflected the agenda of its author, Madeleine Simms, who helped shape the 1967 Abortion Act in her role as General Secretary of the Abortion Law Reform Association. Elsewhere, she wrote about the ‘heavy burden’ facing mothers ‘dragooned’ into providing ‘total care, 24 hours a day, for a lifetime’ for their disabled children.^93^ As Dagmar Herzog has noted, campaigns to expand abortion access in the 1960s and 1970s drew heavily on the assumption that ‘to bear and raise a disabled child would be an especially awful fate.’^94^

These responses reflected the experiences of parents, not their disabled children. Yet of the two audience members who themselves had spina bifida, one described their life as ‘a living hell’ filled with endless surgeries, and both insisted that if they had a child with spina bifida, they’d not want the child to survive.^95^ However, the quality of life was not determined purely by impairment; it was also shaped by living in a society that held profoundly discriminatory attitudes towards disabled people. Later studies with survivors attributed psychological maladjustment and extreme social isolation largely to ‘dependency on others, restricted choices, physical barriers and the adverse reactions of others.’^96^

One approach to the growing awareness of families’ difficulties in caring for disabled children was to advocate for a greater commitment from the state and society to supporting disabled people. By the 1970s, the Disablement Income Group had successfully drawn attention to gaps in welfare provisions for disabled people, though practical gains were limited,^97^ while new organisations like the Union of Physically Impaired Against Segregation developed rights-based campaigning which targeted the social oppression of disabled people.^98^ Yet these campaigns unfolded against discussions on resource allocation within the NHS which questioned the value of disabled lives. Simms, for example, argued that debates about caring for disabled people should not happen in ‘a financial vacuum’, and that neither mothers nor the state should carry the ‘burden’. Claiming that people—presumably Simms meant able-bodied people - were dying, who could survive with appropriate care, Simms argued that ‘certain priorities in healthcare ought to be asserted based on likely quality of life.’^99^ Lambasting the inadequate support offered to parents of disabled children, Glendinning argued that help should be a right, not a favour, but recognised that many continued to see disability as a private, family tragedy.^100^ Parents were not always seen as culpable for what had befallen them; parents of children born with thalidomide-induced limb anomalies, for example, were viewed as victims of a negligent pharmaceutical firm. The situation differed for parents of babies born with spina bifida. When prenatal screening for neural defects was still being rolled out across the NHS,^101^ and knowledge of folate’s role in reducing the incidence of neural defects was limited,^102^ it was impossible for anyone to blame parents for giving birth to babies with spina bifida myelomeningocele. Moreover, while government policy dictated that all children should be actively treated, parents were not asked by doctors to make decisions about whether their child should receive treatment.

However, once the DHSS tacitly endorsed selective non-treatment, and doctors began insisting that parents should decide whether children received treatment, parents were suddenly burdened with the responsibility of making the right ‘moral’ choice. Given the preceding discussion of how flawed the information and consent process parents were increasingly expected to participate in was, the injustice is clear. Many parents may well have leaned towards doctors’ views, deferring to doctors’ authority, and seeking to share the decision-making burden. As we have seen, some parents found the decisions they had made almost unbearable to live with and continued to feel a destructive sense of guilt about their child’s death, whether this was inevitable, preventable, and justified or not, which led in some cases to divorce and acute psychological distress.^103^ Others however sought to defend themselves against guilt by refusing to admit any uncertainty or deviate from asserting that they made the right decision, emphasising either their disabled child’s enjoyment of life, or how their child’s early death had prevented unnecessary suffering.

Louise’s parents fell into the latter of these two categories. While they expressed a desire to spare her pain, they were also unable to see disability as anything other than a disaster which would ruin her life, when set alongside able-bodied existence. As Louise’s father explained, ‘part of our feelings about Louise was… that as she grew up, in comparison with two perfectly normal girls, she’d obviously compare herself with them… We didn’t feel as though we could more or less condemn her to a life… that was so much incomplete in comparison with the other two girls.’ Louise’s mother recalled coming home one evening as her elder two daughters ran around, got undressed and got ready for bed, and ‘it hit me then, you know, that Louise would never be able to do anything like that’.^104^

However, some parents robustly challenged this presentation of disability as something that automatically removed enjoyment and capacity. One mother explained that her daughter had initially fetched and carried for her disabled son, but now simply told her son to get on with it. This mother denied the presenter’s contention that she had made ‘great sacrifices’ to have her son, claiming she had ‘got a great deal out of life’ through him. Emma’s mother wrote in to assert that her daughter ‘has brought more joy in the lives of my husband and me than sorrow’. Louise’s parents, she wrote ‘did right as they saw it, and sometimes when I see Emma outside with no one to play with and the world passing by, it makes me feel very sad. But life is for living, and that is what Emma does, and she enjoys it.’^105^ Another parent was told that their son, James, was going to die, and was given the option of placing him in an institution. They reported that ‘he’s a perfectly…he’s still disabled, he’s partially paralysed from the waist downwards, but he’s a very normal well-adjusted twelve-year-old boy who I’m very glad lived.’ Sheila’s parents had already lost one child to spina bifida when Sheila was born with the same condition. Told that Sheila had a better prognosis than her older sibling, her parents supported active treatment, ‘because I wanted life for Sheila, and life she’s got… she’s happy, she’s cheerful.’ Their discussion of Sheila’s impairments revealed that they found the experience of living with spina bifida to be far more manageable than Garrow presented to new parents. ‘Alright, she’s got the valve, she’s got the loop, she’s paralysed from the waist downwards, but she can walk with callipers, and, you know, she enjoys life.’^106^

## “Thou shalt not kill; but needst not strive officiously to keep alive”

Louise’s case illuminates the obfuscation surrounding the ethics and practices of withholding care from babies born with congenital anomalies. The burden parents felt when making care decisions, and the guilt some experienced after their child’s death, perhaps reflected their understanding that their babies received care that hastened or caused their deaths, as the earlier recollection from Michael’s mother suggests.

The deaths of babies like Louise tended to be explained in two ways: first, that healthcare professionals allowed such babies to die, suggesting that death was largely a foregone conclusion, and those who actively treated babies were interfering unduly. Second, that doctors actively caused these deaths, when they might otherwise not have occurred. Similarly, the care that babies like Louise did and didn’t receive could be presented in different lights. While this varied from case to case, usually babies were not tube-fed where this might otherwise be clinically advised. Typically, healthcare workers claimed to feed such babies on demand, although feeds might be limited, while some babies, like John Pearson, were only given water. Where infection manifested, antibiotics would usually be withheld, and babies were often sedated.

In Louise’s case, Garrow claimed that he sedated Louise to relieve discomfort from her distended bladder, although this distension arose only because the hospital ceased manually expressing Louise’s bladder. However, when describing the case of Damian, another baby who had died under his care, Garrow admitted sedating Damian to hasten his death and minimise the suffering of his mother, Maxine. ‘I felt that it was important that the baby didn’t go on for three months, because of Maxine. Emotionally, she was extremely upset anyway… if it had gone on for three months it would have been unbearable.’^107^ There was undoubtedly some truth in this. Some of the most harrowing accounts from Delight and Goodall’s research came from bereaved parents who believed their child would die rapidly if they were not operated on yet were forced to watch their child slowly decline in health. As one parent recalled: ‘it was a terribly big decision we had to make about surgery. Then, when she didn’t die in a few days, we kept wondering whether we were wrong. It was hard, just waiting for her to die.’^108^

Certainly, heavy sedation explains why babies fed on demand starved, a leading cause of Louise’s death. When asked by the interviewer about their decisions, Louise’s mother replied, ‘we just said that we didn’t want her to have any more operations.’ However, action taken extended beyond this: as Louise’s mother recalled, ‘she carried on having her bottles, but with some sedation in them, and then she just lost her appetite and she just used to have water, and then she just died.’ This explanation was provided when the interviewer asked her, ‘So what did you finally decide to do?’, a question which assigned agency to Louise’s parents. However, Garrow guided them to this course of action, explaining that Louise’s parents ‘agreed with me that if one was going to withhold operations which would be lifesaving, then one should also withhold antibiotics, which are lifesaving.’ ‘I have no brief to kill,’ explained Garrow, ‘but one can refrain from officiously striving to keep alive, and this is what one tries to do.’^109^ Garrow was not the only proponent of selective non-treatment who drew on this phrase to justify his actions.^110^ Often treated by doctors as authoritative ethical guidance akin to the Hippocratic oath, the phrase came from Arthur Hugh Clough’s mid nineteenth-century poem ‘The Latest Decalogue’, a cynical reworking of the ten commandments, where it functioned as a satirical rephrasing of the commandment not to murder. Louise became dehydrated, dying around three weeks after the decision was taken to withhold treatment and introduce sedation. Pushed by the interviewer on the cause of death, Garrow was noncommittal, responding ‘you could say it was a mixture of infection, I think the urine was infected by the end, which we weren’t treating, but it was dehydration and starvation if you like.’^111^

All doctors participating in the programme agreed that the practice of withholding life-sustaining care was widespread and furtive, with little open discussion of methods. Garrow, for example, claimed that ‘a lot’ of his colleagues did not operate or actively treat children born with spina bifida, while noting ‘I can’t say how they do this, and what they do’. Zachary agreed that withholding treatment was widespread, but claimed that patients were killed, not helped to die. He noted that before the introduction of surgical repair, many patients nevertheless survived. ‘Why are they now dying?’ he asked.^112^ Zachary was challenged by presenter Mary Parkinson, who accused doctors of keeping babies alive who would ‘in a natural course of event, have died’. ‘Don’t you think’, she asked him, ‘that as a medical practitioner you have a responsibility to society, to these parents, to do something about that?’^113^ We might now see Zachary a progressive vocal defender of disabled children’s rights. At the time he was sometimes presented as a reactionary, whose rigid adherence to Catholicism compelled him to persist in treating patients with ‘inevitable tragic results’, as Robert Reid, editor of the BBC’s *Horizon*, put it in the late 1970s.^114^

Parkinson may also however have been trying to diffuse Zachary’s contention that Garrow’s actions constituted euthanasia or manslaughter, which could prompt a criminal investigation. Indeed, Braden felt it necessary to clarify in the studio discussion that Garrow was not ‘looking for prosecution to bring the issue to a head.’^115^ Recent histories on the anti-abortion movement reveal how LIFE and the Society for the Protection of Unborn Children (SPUC) campaigned against abortion on the grounds of foetal disability.^116^ However, both organisations also campaigned against selective non-treatment. Indeed Alison Davis, who was born with spina bifida myelomeningocele and led SPUC’s newly established Handicap Division in 1982, later identified watching the *Afternoon Plus* episodes on Louise’s death as the spark that led her down the path to anti-abortion/pro-life activism.^117^ In 1977, SPUC held a press conference to publicise the issue.^118^ The following year, SPUC activists called for an investigation into Margaret Mayell, a paediatric surgeon at Nottingham City Hospital, after she publicly disclosed that she had withdrawn treatment from disabled babies following consultation with parents.^119^ In Mayell’s case, the Director of Public Prosecutions (DPP) decided there was no evidence of criminality after she completed a questionnaire.^120^

Leading figures within SPUC and Life wrote to the DPP and Thames Valley Police shortly after the first programme was transmitted to demand an investigation into Louise’s death, accusing Garrow of manslaughter.^121^ In notes on the case which highlighted the earlier investigation into Mayell, a DPP official concluded that an enquiry into Garrow’s actions was ‘perhaps unfortunately’ necessary, partly because Garrow had directly attributed Louise’s death in the programme to dehydration and starvation, and partly because of the publicity generated by Garrow’s comments, seemingly an allusion to SPUC and Life’s efforts to publicise the case.^122^

While Garrow deemed the 1973 booklet, *Care of the Child with Spina Bifida*, ‘of limited help’ in answer to a series of prepared questions from Thames police,^123^ it did help protect Garrow from any substantive investigation. A DHSS solicitor sent a copy to the DPP, noting that ‘this of course not to say that there is no more up-to-date publication on this subject, but of which at this moment we have no immediate knowledge.’^124^ Garrow also revised his view on the causes of Louise’s death in his answers to Thames police, claiming his original attribution of death to starvation and dehydration was ‘an over simplification’.^125^ This response provided the DPP with a rationale for dropping criminal charges.

## Conclusion

Despite the publicity her case attracted, the wider ramifications of Louise’s life and death appear limited. Undeterred by the police investigation into Louise’s death, or maybe emboldened by the DPP’s decision to drop the case, and apparently committed to promoting the ethics of selective non-treatment, Garrow featured in a near-identical 1981 BBC documentary. *A Loving Thing to Do?* followed three babies under Garrow’s care at High Wycombe, including Nicola, who was born with an encephalocele. Like Louise, Nicola died from starvation and untreated infection three weeks after her birth.^126^ Garrow retired from practice in 1983.

No legal changes were implemented after the *Afternoon Plus* episodes on Louise aired. *Care of the Child with Spina Bifida* remained the official DHSS line into the early 1980s. Consciously opaque, it provided sufficient latitude for different medical approaches while tacitly encouraging doctors to withhold active treatment. It was used repeatedly in response to letters received expressing concern about the treatment of babies with spina bifida.^127^ However, the rollout of prenatal screening and diagnosis for neural tube defects reduced the overall and unanticipated incidence of births of babies with myelomeningocele, significantly curtailing the agonising decisions facing parents of babies born with myelomeningocele. The number of live and still births where spina bifida was present in England and Wales fell from 677 and 166 respectively in 1979, to 236 and 19 respectively in 1986.^128^ Instead, painful decision-making moved to the antenatal period as expectant mothers grappled with the uncertain prognoses revealed by prenatal screening and diagnosis.^129^ In Britain, a growing focus on children’s rights and parents’ responsibilities slowly reshaped guidance on the care of disabled babies. The section titled “Severely malformed infants” in the 1984 edition of the British Medical Association’s *Handbook of Medical Ethics*, for example, opened with a new sentence, “A malformed infant has the same rights as a normal infant.”^130^

Louise’s life and death had a limited impact on professional practice or policy. Yet her story reveals how the new focus on parental participation in decision-making processes left many with an enduring burden of guilt for their child’s death, even if doctors controlled decision-making in practice. Louise’s story, and that of her more famous namesake, also highlights how disabled babies were seen as a threat to the financial and emotional wellbeing of their parents and siblings, and society more widely. While such views were grounded in pervasive disablist attitudes, babies were particularly vulnerable because many commentators refused to see them as people entitled to the same rights as older children and adults. Singer and Kuhse, for example, while rejecting euthanasia for disabled adults, supported killing disabled newborn babies because their lack of self-awareness or sense of the future meant they could not be classed as people, and therefore did not have ‘an inherent right to life’.^131^ We can at least as historians attempt to reinsert them as subjects into a period when their rights, personhood and lives were called into question.

## Footnotes

1 Robert Edwards papers, *Churchill Archive Centre*, Cambridge; Lesley Brown papers, Bristol Archives, Bristol.

2 Janet Golden, *Babies Made us Modern: How Infants Brought America into the Twentieth Century* (Cambridge: Cambridge University Press, 2018).

3 Duncan Wilson, *The Making of British Bioethics* (Manchester: Manchester University Press, 2014).

4 See six papers on the history of the first IVF birth by Kay Elder and Martin H. Johnson, published as a symposium in *Reproductive BioMedicine and Society*, 2015, *1*, 3–70.

5 Robert G. Edwards, ‘The Bumpy Road to Human *In Vitro* Fertilization’, *Nature Medicine,* 2002, *7*, 1091–94; Martin Powell, *Louise Brown: My Life as the World’s First Test-Tube Baby* (Bristol: Bristol Books, 2015), 47–63; Lesley and John Brown with Sue Freeman, *Our Miracle Called Louise: A Parents’ Story* (New York and London: Paddington Press Ltd, 1979), 152–84.

6 For example, Human Fertilisation & Embryology Authority, ‘Celebrating 40 years of IVF’, accessed October 6, 2023, https://www.hfea.gov.uk/celebrating-40-years-of-ivf/.

7 For example, Science Museum, ‘Louise Joy Brown I The world’s first IVF baby’, posted July 11, 2018, https://www.youtube.com/watch?v=Voy-l403ynE.

8
https://tvark.org/afternoon-plus and Monday 15th January 1979 ITV Granada - Adverts - Take My Wife - After Noon Plus - Bernard Braden (youtube.com). Kaleidoscope, which coordinates ITV’s Raiders of the Lost Archives campaign, lists both episodes as missing: https://www.tvbrain.info/tv-archive?showname=After±noon±Plus&type=lostshow. All sites accessed July 26, 2024.

9
*Afternoon Plus* transcript, 15 January 1979, and *Afternoon Plus* transcript, 22 January 1979, DPP 2/6827, Doctor Hugh Garrow: charges considered for manslaughter of an infant at High Wycombe General Hospital, Buckinghamshire. No action case, The National Archives (TNA), Kew. In the National Archives’ catalogue, the prefix DPP indicates records inherited and created by the Director of Public Prosecutions.

10 Josh Halliday, ‘Mother Tells Lucy Letby Murder Trial she Heard Baby’s ‘Horrendous’ Screams’, *The Guardian*, November 14, 2022, https://www.theguardian.com/uk-news/2022/nov/14/mother-tells-lucy-letby-trial-she-heard-babys-horrendous-screams.

11 Golden, *Babies Made us Modern*, 3.

12 Harry Hendrick, ‘The Child as a Social Actor in Historical Sources: Problems in Identification and Interpretation’, in Pia Christensen and Allison James, eds, *Research with Children: Perspectives and Practices*, (London and New York: Falmer Press, 2000), 36–61; 43.

13 Martin H. Johnson, ‘Robert Edwards: The Path to IVF’, *Reproductive Biomedicine Online*, 2011, *23*, 245–62.

14 Martin H. Johnson *et al*, ‘Why the Medical Research Council Refused Robert Edwards and Patrick Steptoe Support for Research on Human Conception in 1971’, *Human Reproduction*, 2010, *25*, 2157–74.

15 Robert Edwards and Patrick Steptoe, *A Matter of Life: The Sensational Story of the World’s First Test-Tube Baby* (London: Sphere Books Ltd, 1980), 124–25.

16 Powell, *Louise Brown*, 8.

17 On the development of prenatal screening technologies, including amniocentesis, see Ilana Löwy, *Imperfect Pregnancies: A History of Birth Defects and Prenatal Diagnosis* (Baltimore: John Hopkins University Press, 2017).

18 Edwards and Steptoe, *A Matter of Life*, 112.

19 Brown with Freeman, *Our Miracle Called Louise*, 118.

20 Powell, *Louise Brown*, 7, 9.

21
*Ibid*., 37.

22 Katharine Dow, ‘Looking into the Test Tube: The Birth of IVF on British Television’, *Medical History*, 2019, *63*, 189–208, 197.

23 Tom Shakespeare and Nicholas Watson, ‘The Social Model of Disability: An Outdated Ideology?’ *Research in Social Science and Disability*, 2001, *2*, 9–28, 22.

24 N. Akalan, ‘Myelomeningocele (Open Spina Bifida) – Surgical Management,” in J. D. Pickard *et al*, eds, *Advances and Technical Standards in Neurosurgery Vol. 37* (Wein: Springer-Verlag, 2011), 113–37, 120.

25 Franc D. Ingraham and Henry Swan, ‘Spina Bifida and Cranium Bifidum. I. A Survey of Five Hundred and Forty-Six Cases’, *The New England Journal of Medicine*, 1943, *228*, 559–63.

26 Joshua S. Baru, *et al.*, ‘John Holter’s Shunt’, *Journal of the American College of Surgeons*, 2001, *192*, 79–85.

27 Kenneth L. Tyler, ‘Chapter 28: A History of Bacterial Meningitis’, in Michael J. Aminoff, François Boller and Dick F. Swaab, eds, *Handbook of Clinical Neurology Volume 95* (Edinburgh: Elsevier, 2009), 417–33.

28 Robert Zachary, *Surgeon to a Child: Memoirs of a Paediatric Surgeon* (Burwood: Foundation Genesis, 1987), 46–51.

29 W. John W. Sharrard, *et al*., ‘A Controlled Trial of Immediate and Delayed Closure of Spina Bifida Cystica’, *Archives of Disease in Childhood*, 1963, *30*, 18–22.

30 ‘Spina Bifida’, briefing note for conference held 17 July, 1964, MH 159/20, congenital abnormalities (especially spina bifida and hydrocephalus): development of treatment and facilities available, 1963–1965, TNA. The National Archives’ catalogue uses the prefix MH to indicate records created or inherited by the Ministry of Health and successors.

31 Anne Hardy, *Medicine and Health in Britain since 1860* (Basingstoke: Palgrave, 2001), 155.

32 Letter from Robert Zachary to George Godber, 24 April, 1963, MH 159/20; letter and attached memorandum from Peter Rickham to George Godber, 23 July, 1964, MH 159/20; letter from Peter Rickham to Albertine Winner, 22 October, 1965, MH 159/21, congenital abnormalities (especially spina bifida and hydrocephalus): development of treatment and facilities available, 1965, TNA.

33 Note dated February 1969, MH 159/22, congenital abnormalities (especially spina bifida and hydrocephalus): development of treatment and facilities available, 1967–71, TNA.

34 Central Health Services Council and Scottish Health Services Council, *Surgery for the Newborn: Report of the Joint Sub-Committee of the Standing Medical Advisory Committees* (London: HMSO, 1968), 1–2.

35 J. Lorber, ‘Results of Treatment of Myelomeningocele: An Analysis of 524 Unselected Cases, with Special Reference to Possible Selection for Treatment’, *Developmental Medicine and Child Neurology*, 1971, *13*, 279–303, 279.

36 Standing Medical Advisory Committee for the Central Health Services Council, the Secretary of State for Social Services, and the Secretary of State for Wales, *Care of the Child with Spina Bifida* (London and Cardiff: DHSS and Welsh Office, 1973).

37
*Care of the Child with Spina Bifida*, p. 4.

38 Lisa J. Pruitt, ‘Living with Spina Bifida: A Historical Perspective’, *Pediatrics*, 2012, *130*, 181–83.

39 Lorber, ‘Results of Treatment’, 280.

40 M. F. Robards, G. G. Thomas, L. Rosenbloom, ‘Survival of Infants with Unoperated Myeloceles’, *British Medical Journal*, 1975, *4*, 12–13.

41 Eileen Delight and Janet Goodall, ‘Love and Loss. Conversations with Parents of Babies with Spina Bifida Managed Without Surgery, 1971-1981’, *Developmental Medicine and Child Neurology*, 1990, *32*, supplement 61, 1–58, 24. 51 percent of these babies were prescribed sedatives: 28–29.

42 R. B. Zachary, ‘Life with Spina Bifida’, *British Medical Journal*, 1977, *2*, 1460–62; 1461.

43 John M. Freeman, ‘Life with Spina Bifida: The Role of Quality of Life in Decision-Making’, *The Journal of Pediatrics*, 2013, *162*, 894–95.

44 Norman Guthkelch, ‘Arthur Norman Guthkelch: An Autobiographical Note’, in ‘Special Edition: on “Shaken Baby Syndrome”’, *Argument and Critique*, ISSN 2059-7606, accessed July 7, 2021. https://www.argumentcritique.com/uploads/1/0/3/1/10317653/norman.pdf.

45 Jilly Boyce Kay, ‘“The Sunday Times Among Them”: Good Afternoon! and the Gendering of Afternoon Television in the 1970s,’ *Critical Studies in Television*, 2014, 9, 74–93.

46 TV Times January 13th–19th 1979 (London Region), 47; accessed via https://www.themagazinearchive.com/; Jeremy Potter, *Independent Television in Britain Volume 4: Companies and Programmes 1968-80* (Macmillan: Basingstoke, 1990), 7.

47 Boyce Kay, ‘“The Sunday Times’”, 75.

48 Ibid, 77.

49 Ibid, 77.

50 Bernard Braden, *The Kindness of Strangers* (London: Hodder and Stoughton, 1990).

51 Anne Karpf, *Doctoring the Media: The Reporting of Health and Medicine* (Routledge: London, 1988), 57–63.

52 Elizabeth Toon, ‘The Machinery of Authoritarian Care: Dramatising Breast Cancer Treatment in 1970s Britain,’ *Social History of Medicine*, 2014, *27*, 557–76.

53 Ayesha Nathoo, *Hearts Exposed: Transplants and the Media in 1960s Britain* (Basingstoke: Palgrave Macmillan, 2009), 86–110. This debate was groundbreaking in using television to explore medical controversy, and by featuring identifiable doctors alongside patients. Guidance against indirect advertising had previously deterred doctors from appearing unless anonymised.

54 Other examples include: *Controversy*, producer Dominic Flessati, aired in BBC2, 27 February 1975; *Man Alive: A Loving Thing to Do?*, producer Louise Panton, aired on BBC2, 26 February 26 1981; *Horizon: A Small Imperfection*, producer Robert Reid, aired on BBC1, 25 February 1975; Ian Kennedy, *The Defect*, producer David Paterson, aired on BBC Radio4, 24 October and 31 Oct 1978; *Dilemmas: 3: Life - at Any Cost?*, producer Chris Jelley, aired on BBC1, 21 May 1978; *ITV Playhouse: Lindsay*, producer John Bowen, aired on ITV Thames, 22 July 1980; *Jaywalking: Live or Let Die*?, producers Graham Mole and Sam Fairhill, aired on ITV, 1 March 1981.

55 Karpf, *Doctoring the Media,* 59–63.

56
*Afternoon Plus*, 15 January 1979.

57 D. M. T. Gairdner, ‘Dermod De La Chevallerie MacCarthy’, accessed July 19, 2021, https://history.rcplondon.ac.uk/inspiring-physicians/dermod-de-la-chevallerie-maccarthy.

58 C. H. Cheetham, ‘Donald Hugh Garrow’, accessed March 1, 2024, https://history.rcplondon.ac.uk/inspiring-physicians/donald-hugh-garrow.

59 Donald H. Garrow, ‘Special Care without Separation: High Wycombe, England’, in J.A. Davis, M.P.M. Richards and N.R.C. Robertson, eds, *Parent-Baby Attachment in Premature Infants* (Kent: Croom Helm, 1983), 223–31, 224.

60 Nicola’s case featured in *Man Alive, A Loving Thing to Do?*

61 R. B. Zachary, ‘Editorial’, *Link: The Journal of the Association for Spina Bifida and Hydrocephalus Limited*, 1968, *1*, 3.

62 Zachary, ‘Life with Spina Bifida’.

63 Zachary, *Surgeon to a Child*, 129.

64 Jameel Hampton, *Disability and the Welfare State in Britain: Changes in Perception and Policy, 1948-79* (Bristol: Policy Press, 2016), 163.

65 Quoted in Marjorie Wallace, ‘It Had Been a Cruel Lottery, Some Babies Resembling Jumbled Jigsaws’, *The Sunday Times,* May 6, 2012, 18.

66 Hampton, *Disability*, 168.

67 See Claire Sewell, ‘“If One Member of the Family is Disabled the Family as a Whole is Disabled”: Thalidomide Children and the Emergence of the Family Carer in Britain, c.1957-1978’” *Family and Community History*, 2015, *18*, 37–52.

68 Neil McIntosh, ‘The Early Years. Ethics and Terminal Care by Paediatricians and the Ethics Committee: From Leonard Arthur to Withholding and Withdrawing Life-Sustaining Medical Care’, in Alan Croft and Keith Dodds, eds, *From an Association to a Royal College: The History of the British Paediatric Association and the Royal College of Paediatrics and Child Health 1988-2016* (Cham: Springer, 2017), 110–13, 110.

69
*Ibid*., 110–11.

70 British Medical Association, *The Handbook of Medical Ethics* (London: British Medical Association, 1980), 29.

71 Wilson, *The Making of British Bioethics*, 64–129.

72 Carolyn Faulder, *Whose Body Is It? The Troubling Issue of Informed Consent* (London: Virago, 1985), 118–19.

73
*Ibid*., 41–42.

74
*Afternoon Plus*, 22 January 1979

75
*Ibid*.

76 Caroline Glendinning, *Unshared Care: Parents and Their Disabled Children* (London: Routledge and Kegen Paul, 1983), 22.

77
*Ibid*., 121.

78
*Afternoon Plus*, 22 January 1979.

79 Delight and Goodall, ‘Love and Loss’, 12.

80
*Afternoon Plus*, 22 January 1979.

81
*Afternoon Plus*, 15 January 1979.

82 Garrow, ‘Special Care Without Separation’, 230.

83
*Afternoon Plus*, 22 January 1979.

84 Annemarie Mol, *The Logic of Care: Health and The Problem of Patient Choice* (London: Routledge, 2008).

85 Delight and Goodall, ‘Love and Loss’, 4.

86 Mol, *The Logic of Care*, 7.

87
*Afternoon Plus*, 22 January 1979.

88 Delight and Goodall, ‘Love and Loss’, 22.

89 Sewell, ‘“If One Member of the Family”’.

90
*Afternoon Plus*, 22 January 1979.

91
*Ibid*.

92 Madeleine Simms, ‘Informed Dissent: The Views of Some Mothers of Severely Mentally Handicapped Young Adults’, *Journal of Medical Ethics*, 1986, *12*, 72–74.

93 Madeleine Simms, ‘Care of the Severely Handicapped’, Journal of Medical Ethics, 1988, *14*, 54–55; 54.

94 Dagmar Herzog, *Unlearning Eugenics: Sexuality, Reproduction and Disability in Post-Nazi Europe* (Wisconsin: University of Wisconsin Press, 2018), 16.

95
*Afternoon Plus*, 22 January 1979.

96 Michael Hirst, ‘Patterns of Impairment and Disability related to Social Handicap in Young People with Cerebral Palsy and Spina Bifida’, *Journal of Biosocial Science*, 1989, *21*, 1–12; 11.

97 Hampton, *Disability and the Welfare State*, 181–244.

98 Vicky Long, ‘Disability and Disabled People in 20^th^ Century Britain’, in Nicole Robertson, John Singleton and Avram Taylor, eds, *20th Century Britain: Economic, Cultural and Social Change* (Abingdon: Routledge, 2023), 202–15, 211.

99 Simms, ‘Care of the Severely Handicapped’, 54.

100 Glendinning, *Unshared Care*, 237–38.

101 Löwy, *Imperfect Pregnancies*, 106–7.

102 Salim Al-Gailani, ‘Making Birth Defects ‘Preventable’: Pre-Conceptional Vitamin Supplements and the Politics of Risk Reduction’, *Studies in History and Philosophy of Biological and Biomedical* Sciences, 2014, *47*, 278–89.

103 Goodall and Delight, ‘Love and Loss’.

104
*Afternoon Plus*, 15 January 1979.

105
*Afternoon Plus*, 22 January 1979.

106
*Ibid*.

107
*Afternoon Plus*, 22 January 1979.

108 Delight and Goodall, ‘Love and Loss’, 35.

109
*Afternoon Plus*, 15 January 1979.

110 See, for example, L. Haas, ‘Ethical and Social Aspects of Treatment of Spina Bifida’, *The Lancet*, 1968, *292*, 974.

111
*Afternoon Plus*, 15 January 1979.

112
*Afternoon Plus*, 22 January 1979.

113
*Ibid.*

114 Robert Reid, *My Children, My Children. Life Before and After Birth: An Account of Some Recent Developments* (London: BBC, 1976), 27. This was an adaptation of a three part *Horizon* series, the third of which, *A Small Imperfection,* dealt with spina bifida, and aired on BBC One on 21 December 1976 at 23:10.

115
*Afternoon Plus*, 22 January 1979.

116 Olivia Dee, *The Anti-Abortion Campaign in England, 1966-1989* (Routledge: New York and London, 2020); Sally Sheldon, Gayle Davis, Jane O’Neill and Clare Parker, *The Abortion Act of 1967: A Biography of a UK Law* (Cambridge: Cambridge University Press, 2023).

117 Alison Davis, *From Where I Sit: Living with Disability in an Able Bodied World* (Triangle: London, 1989), 91.

118 Anonymous, ‘Hundreds of Babies Killed in Hospitals’, *Daily Mail*, December 29, 1977, 13.

119 Anonymous, ‘Police Asked to Inquire into Baby Deaths’, *The Times*, January 10, 1978, 3.

120 Notes made by R. E. Glenister, 27 February 1979, DPP 2/6827. Mayell’s name is redacted, but cross-­referencing newspaper coverage reveals that the allegations were raised about her. See ‘Margaret Joyes Mayell and Family’, *Leatherhead & District Local History Society Newsletter*, May 2019, *35*, https://leatherheadhistoryarchive.org/wp-content/uploads/2019/10/LDLHS-Newsletter-2019-March.pdf.

121 Letter from John Smeaton to DPP, 15 January 1979 and letter from Nuala Scarisbrick to DPP, 16 January 1979, DPP 2/6827.

122 Notes by Richard E. Glenister, 27 February 1979, DPP 2/6827.

123 Answers of Dr Garrow to questions handed to him on 2.4.1979, 14 May 1979, DPP 2/6827.

124 Letter from I.C.N. Hamilton to Richard Glenister, 28 March 1979, DPP 2/6827.

125 Answers of Dr Garrow.

126 Man Alive, *A Loving Thing to Do?*

127 As seen in DHSS replies to letters expressing concern about the treatment of spina bifida babies in MH 152/138.

128 On screening, see Löwy, *Imperfect Pregnancies*, 106–7. Figures from Written Answer, 7 May 1987, MH 152/138.

129 See Barbara Katz-Rothman, *The Tentative Pregnancy: Prenatal Diagnosis and the Future of Motherhood* (Harmondsworth: Viking Penguin, 1986).

130 British Medical Association, *The Handbook of Medical Ethics* (London: British Medical Association, 1984), 63.

131 Helga Kuhse and Peter Singer, *Should the Baby Live? The Problem of Handicapped Infants* (Oxford: Oxford University Press, 1985), 192.

## Author Biography

Vicky Long trained and worked within several of the UK’s research centres for the history of medicine/health—Warwick, Manchester and Glasgow’s CSHHH—before joining Newcastle in 2018 as Senior Lecturer in Twentieth-Century British History. At Newcastle, she co-founded and co-leads Newcastle’s Medical Humanities Network. She has published articles, book chapter and co-edited books on the history of occupational health, disability, work and mental health. She is the author of Destigmatising Mental Illness? Professional Politics and Public Education in Britain, 1870–1970 and The Rise and Fall of the Health Factory: The Politics of Industrial Health in Britain, 1914–1960. Vicky is currently writing a book which uses spina bifida as a case study to examine the impact of emerging technocratic approaches to medicine in late twentieth-century Britain on disabled people.

